# Comparative genomic analysis of *Campylobacter jejuni *associated with Guillain-Barré and Miller Fisher syndromes: neuropathogenic and enteritis-associated isolates can share high levels of genomic similarity

**DOI:** 10.1186/1471-2164-8-359

**Published:** 2007-10-05

**Authors:** Eduardo N Taboada, Alex van Belkum, Nobuhiro Yuki, Rey R Acedillo, Peggy CR Godschalk, Michiaki Koga, Hubert P Endtz, Michel Gilbert, John HE Nash

**Affiliations:** 1 Institute for Biological Sciences, National Research Council, 100 Sussex Drive, Ottawa, Ontario, K1A 0R6, Canada; 2 Department of Medical Microbiology and Infectious Diseases, Erasmus MC, University Medical Center Rotterdam, 3015 GD Rotterdam, The Netherlands; 3 Department of Neurology and Research Institute of Neuroimmunological Diseases, Dokkyo Medical University School of Medicine, Shimotsuga, Tochigi 321-0293, Japan

## Abstract

**Background:**

*Campylobacter jejuni *infection represents the most frequent antecedent infection triggering the onset of the neuropathic disorders Guillain-Barré syndrome (GBS) and Miller Fisher syndrome (MFS). Although sialylated ganglioside-mimicking lipo-oligosaccharide (LOS) structures are the strongest neuropathogenic determinants in *C. jejuni*, they do not appear to be the only requirement for a neuropathic outcome since strains capable of their production have been isolated from patients with uncomplicated cases of enteritis. Consequently, other pathogen and/or host-related factors contribute to the onset of neurological complications. We have used comparative genomic hybridization to perform a detailed genomic comparison of strains isolated from GBS/MFS and enteritis-only patients. Our dataset, in which the gene conservation profile for 1712 genes was assayed in 102 strains, including 56 neuropathogenic isolates, represents the largest systematic search for *C. jejuni *factors associated with GBS/MFS to date and has allowed us to analyze the genetic background of neuropathogenic *C. jejuni *strains with an unprecedented level of resolution.

**Results:**

The majority of GBS/MFS strains can be assigned to one of six major lineages, suggesting that several genetic backgrounds can result in a neuropathogenic phenotype. A statistical analysis of gene conservation rates revealed that although genes involved in the sialylation of LOS structures were significantly associated with neuropathogenic strains, still many enteritis-control strains both bear these genes and share remarkable levels of genomic similarity with their neuropathogenic counterparts. Two capsule biosynthesis genes (Cj1421c and Cj1428c) showed higher conservation rates among neuropathogenic strains compared to enteritis-control strains. Any potential involvement of these genes in neuropathogenesis must be assessed. A single gene (HS:3 Cj1135) had a higher conservation rate among enteritis-control strains. This gene encodes a glucosyltransferase that is found in some of the LOS classes that do not express ganglioside mimics.

**Conclusion:**

Our findings corroborate that neuropathogenic factors may be transferred between unrelated strains of different genetic background. Our results would also suggest that the failure of some strains isolated from uncomplicated cases of enteritis to elicit a neuropathic clinical outcome may be due to subtle genetic differences that silence their neuropathogenic potential and/or due to host-related factors.

The microarray data has been deposited in NCBI's Gene Expression Omnibus under accession number GSE3579.

## Background

*Campylobacter jejuni *infection is a leading cause of acute bacterial gastroenteritis worldwide [1]. The widespread dissemination of *C. jejuni *is largely attributed to its transmission to humans by consumption of contaminated food sources frequently colonized by the bacteria. Clinical symptoms vary from mild to severe gastroenteritis to more complex and serious extraintestinal diseases, including the neuropathic disorders Guillain-Barré syndrome (GBS) or its variant Miller Fisher syndrome (MFS) [2].

GBS and MFS are acute neuropathies thought to result from a transient humoral immune response against host gangliosides in peripheral nerves [3]. GBS is the most common form of acute flaccid paralysis with incidence rates of up to 4 per 100, 000 [4]. MFS, a variant of GBS, is characterized by acute ophthalmoplegia and ataxia. The onset of these syndromes is often preceded by infectious illness and *C. jejuni *is the most frequent infectious agent. 30% of GBS cases and 20% of MFS cases are preceded by an infection of *C. jejuni *within three weeks prior to the onset of neurological symptoms [5-7]

A number of studies have sought to examine the population structure of GBS/MFS strains in the search for genetic commonalities that could account for a neuropathogenic phenotype. Initial surveys showing an over-representation of serotypes HS:19 and HS:41 among GBS strains [8-11], raised the possibility that GBS strains comprise a clonal lineage with unique virulence factors associated with GBS. For example, a high proportion of HS:19 isolates was obtained from GBS patients in countries such as Japan, despite the low prevalence of this serotype among Japanese enteritis cases, suggesting that enteritis patients infected with a strain of HS:19 serotype have an increased risk of developing GBS [8]. A similar association has been observed between Japanese MFS-related *C. jejuni *strains and the HS:2 serotype [10]. Subsequent studies, however, have shown substantial genetic heterogeneity in other collections of neuropathogenic strains [12-14].

Lipo-oligosaccharide (LOS) is one of the most important cell-surface structures expressed by *C. jejuni*, and strains associated with neuropathies express ganglioside-like LOS structures [15,16]. The development of GBS/MFS following *C. jejuni *infection is thought to be related to molecular mimicry between ganglioside-like moieties on certain *Campylobacter *LOS classes and ganglioside epitopes on neural tissue [17], with cross-reacting anti-LOS antibodies ultimately leading to nerve damage [18]. The majority of patients with GBS subsequent to *C. jejuni *enteritis develop autoantibodies that react to GM1 or GD1a gangliosides [19,20] whereas MFS patients develop anti-GQ1b antibodies [5,21]. The LOS from GBS- and MFS-associated *C. jejuni *have been shown to induce anti-GM1 and anti-GQ1b antibodies in rabbits [22]. Moreover, sensitization of rabbits with GM1-like LOS of *C. jejuni *isolated from a GBS patient has resulted in a disease model of GBS [23].

Among the three classes of *C. jejuni *LOS locus (A, B, and C) initially characterized by Gilbert *et al*. [24], the majority of HS:19 isolates harbour a Class A LOS locus, a gene cluster implicated in the expression of ganglioside mimics [25,26]. The A-class LOS carries the *cst-II *gene which, first isolated from the GBS-associated strain OH4384, encodes a bifunctional sialyltransferase capable of transferring sialic acid to either a terminal galactose residue or to a terminal sialic acid residue, resulting in linkages that lead to the production of ganglioside mimics [27]. Taken together, both *cst-II *and the Class A locus are currently the strongest known determinants of GBS [25,26,28-30] Recent studies using knockout mutants of *C. jejuni *and a mouse model have demonstrated the necessity of *cst-II *and of a related sialylation pathway gene (*orf10 *or *neuA1*, encoding a CMP-NeuAc synthetase) in the induction of anti-ganglioside antibodies [25].

Although anti-LOS cross-reactive antibodies are a major component of the development of *C. jejuni *induced GBS and MFS, the complete mechanism is not fully understood. Similarly, the extent to which microbial and/or host factors contribute to the development of an anti-glycolipid response and neurological symptoms remains a point of debate [31]. Cases have been described in which GBS patients with *C. jejuni *infections fail to display ganglioside sero-reactivity, raising the possibility that other peripheral nerve antigens are the targets in these patients [32]. Furthermore, ganglioside-like structures have been found in *C. jejuni *isolates from enteritis patients without GBS or MFS [33]. Recent work aiming to characterize the LOS locus of GBS/MFS-associated *C. jejuni *strains also suggests that there is a strong, but incomplete, correlation between neuropathogenic strains and an A or B-class LOS locus [25,26,29]. Although neuropathogenic strains that carry a non-A/B locus may be less prevalent, their existence raises the possibility that other factors (pathogen and/or host-related) may contribute to the onset of neurological complications. We have performed a systematic search for additional pathogen-related GBS/MFS-associated factors by performing a detailed genetic comparison of strains isolated from GBS/MFS patients, and from uncomplicated cases of enteritis (i.e. "enteritis-control strains"), by means of microarray-based Comparative Genomic Hybridization (CGH). We present here the results of this comprehensive comparative genomic survey.

## Results and discussion

### GBS/MFS strains are genomically heterogeneous

**Figure 1:**
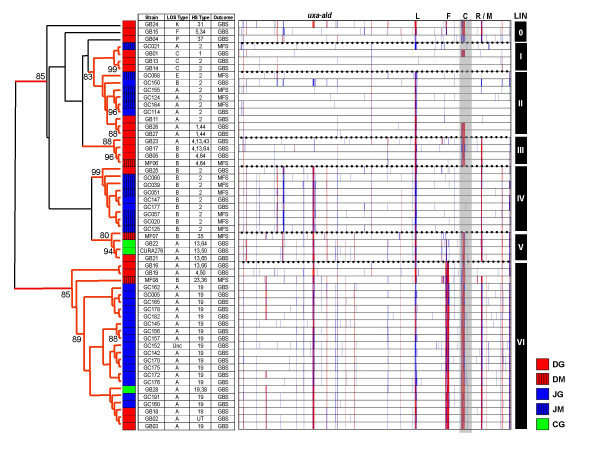
**CGH profiles for the 56 neuropathogenic strains in the combined dataset**. The 56 neuropathogenic strains analyzed for this study show substantially different genetic backgrounds, although most appear to belong to one of six major lineages (LIN). Three strains show unique gene conservation profiles and fail to cluster robustly with any major lineage. Branches with greater than 75% bootstrap support are shown in red. Although data is displayed including capsular genes (gray box), these genes were removed during cluster analysis to avoid biasing results. Highly divergent/Absent genes shown in red; Moderately Divergent genes are shown in blue. Legend: Hypervariable loci (L – LOS locus; F – flagellar modification locus; C – capsular locus; R/M – restriction-modification locus); Strain sets (DG: Dutch GBS; DM: Dutch MFS; JG: Japanese GBS; JM: Japanese MFS; CG: Curaçao GBS).

Previous studies have suggested the heterogeneous nature of neuropathogenic *C. jejuni *strains [12,34]. We compared the CGH profiles of the 56 neuropathogenic strains in our dataset and cluster analysis confirms substantial genomic heterogeneity among the strains studied (Figure 1). However, the results also suggest the presence of several lineages distinguishable from one another based on differences among known hypervariable loci [35].

Clusters I and II are comprised of strains with very little genetic divergence with respect to the genome strain NCTC 11168. Cluster I includes two GBS strains (GB13 and GB14) that show among the lowest levels of divergence observed thus far with respect to the genome strain NCTC 11168. The strains in Cluster II, which include GB11 [36], are very similar to NCTC 11168, albeit with a divergence compared to that strain's C-class LOS locus. Although the bulk of the strains in clusters I and II are of the HS:2 serotype (10 of 13), three strains (GB01, GB26, and GB27) are non-HS:2. Cluster III is comprised of 4 of the 5 HS:4-complex strains in the entire dataset. Cluster IV is largely comprised of Japanese HS:2 strains harbouring significant divergences with respect to NCTC 11168 and also includes a genetically similar Dutch strain (GB25). Cluster V is comprised of neuropathogenic strains from the "Curaçao cluster", a genetically homogeneous group of enteritis and GBS strains from Curaçao that also includes the Dutch GBS/MFS isolates GB21 and MF07. Cluster VI, which at 23 strains is also the largest, includes all HS:19 strains in the dataset regardless of disease outcome or geographical source. All strains in the cluster show a high degree of genomic homogeneity with respect to one another despite the cluster containing a small number of non HS:19 strains.

**Figure 2:**
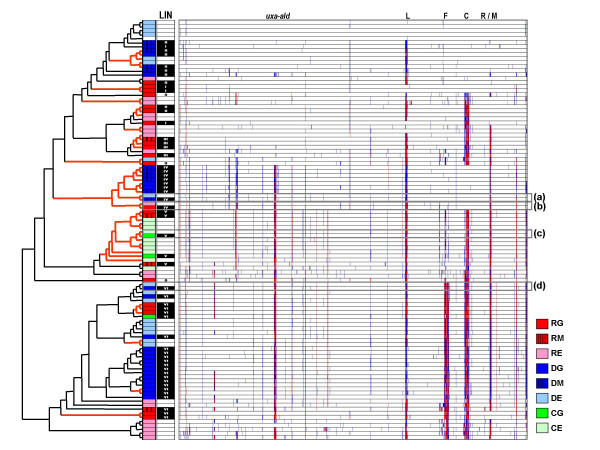
**CGH profile-based clustering of 102 strains included in this study**. Although cluster analysis of 56 neuropathogenic strains produced 6 major lineages, inclusion of the 46 enteritis-control strains shows that these lineages are not exclusively comprised of neuropathogenic strains. Both types of strains can show substantial similarities in genomic background, which includes similarities at several hypervariable regions. The lineage (LIN) of the 56 neuropathogenic strains is shown. Highly similar enteritis-control/neuropathogenic strain pairs (boxes a through d) are shown in expanded form in Figure 3. Legend: Hypervariable loci (L – LOS locus; F – flagellar modification locus; C – capsular locus; R/M – restriction-modification locus); Strain sets (DG: Dutch GBS; DM: Dutch MFS; DE: Dutch enteritis; JG: Japanese GBS; JM: Japanese MFS; JE: Japanese enteritis; CG: Curaçao GBS; CE: Curaçao enteritis).

Although most neuropathogenic strains appear to form part of 6 major genomic lineages, cluster analysis of the 102 strains in our survey shows that every major lineage present in the dataset includes both neuropathogenic and enteritis-only strains (Figure 2). Thus, there appears to be no lineages comprised exclusively of either neuropathogenic or enteritis-control strains. Due to the large number of HS:2 and HS:19 strains in our combined dataset, we performed all cluster analysis after removal of genes from the capsular polysaccharide locus (CPS), to remove any possible bias imparted by the expected differences at this locus. Thus, clusters predominantly composed of strains from serotypes HS:2 (clusters II and IV) and HS:19 (cluster VI) are based on genomic similarities at loci other than the CPS and are likely indicative of clonality among these strains.

### Statistical comparison of gene conservation rates in neuropathogenic and enteritis-control strains

In order to uncover genes associated with neuropathogenic potential, we selected a representative set of neuropathogenic and enteritis-control strains and compared the "absence rate" for each gene in the microarray in both groups of strains. Enteritis-control and neuropathogenic isolates were selected so as to represent the various lineages in our dataset and so as to minimize the effect of the unequal distribution of isolates in each lineage. After analyzing the gene content data for neuropathogenic and enteritis-control isolates, 20 genes were found to have >15% difference in absence rates. Of these, only six genes had statistically significant differences in the absence rate between both groups (Table 1). We found lower absence rates for three markers associated with A/B-class LOS loci and GBS (*cgtA*, *neuA1*, *orf11*) among neuropathogenic strains and although, unexpectedly, our data did not appear to show a significant association for the important GBS marker *cst-II*, we can attribute this to a flaw with the corresponding probe in our microarray. Of the more than 1700 genes tested in our CGH survey, only two additional genes (Cj1421c, Cj1428c or *fcl*) had significantly lower absence rates among neuropathogenic strains compared to the enteritis-control strains. A single gene (HS:3 Cj1135) had a significantly lower absence rate among enteritis-control strains (Table 1).

**Table 1:** Genes with absence rates that differ between enteritis-control strains compared to neuropathogenic strains

**Gene name**	**Proposed function**	**Neuropathogenic strains **(n = 32)		**Enteritis-control strains **^**1 **^(n = 32)		**p-value**^**2**^
		
		**Absent**	**Absence rate (%)**	**Absent**	**Absence rate (%)**	
Cj1421c	Capsule biosynthesis ^3^	0	0	7	21.9	0.0108
*fcl *(Cj1428c)	Capsule biosynthesis ^3^	9	28.13	18	56.3	0.0420
Cj1135 ^4^	One-domain glucosyl transferase ^3^	32	100	26	81.3	0.0242
*cgtA *^5^	*N*-acetyl galactosaminyl transferase	3	9.37	17	53.13	0.0003
*neuA1 *^5^	CMP-NeuNAc synthetase,	3	9.37	14	43.75	0.0038
*orf11 *^5^	Sialic acid acetyl transferase	3	9.37	13	40.63	0.0081

^1 ^Due to the smaller sample size of enteritis-control strains assayed using the newer version of our microarray, 8 enteritis-control strains from unrelated strain collections were randomly selected and included in this group.

^2 ^p-value based on 2-tailed Fisher's Exact Test

^3 ^Proposed function obtained from [48]

^4 ^Gene from strain ATCC 43431 (HS:3 type strain)

^5 ^Gene from strain ATCC 43446 (HS:19 type strain)

### Neuropathogenic and enteritis-control strains can share remarkable levels of genetic similarity

Results from cluster analysis of the CGH data showed several instances in which a neuropathogenic strain and an enteritis-only strain clustered together with very high similarity in genomic profiles. The gene content of these strains was assessed using previously defined thresholds [37] and none of these strain pairs were found to show obvious differences in gene content in the known neuropathogenic markers associated with A/B-class LOS loci. In order to determine whether any additional differences in gene content could be correlated to differences in clinical outcome, four such strain pairs were chosen for a subsequent high-resolution comparison of CGH profiles.

**Figure 3:**
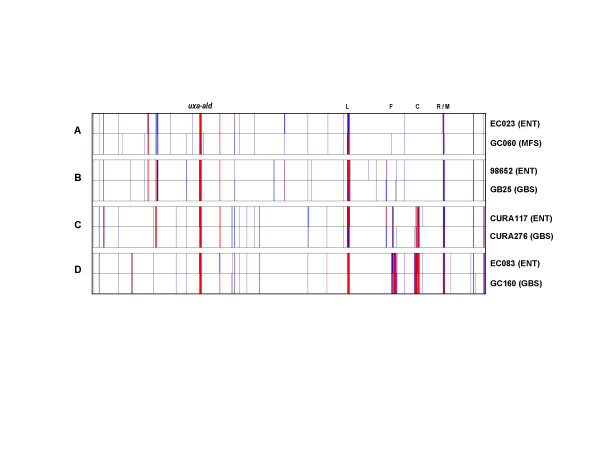
**Gene conservation profiles from closely related pairs of neuropathogenic and enteritis-control strains**. Strain pairs were analyzed separately to look for any potential genetic differences that could be related to differences in clinical outcome. Although each strain pair showed subtle differences in CGH profiles, none of these was common across the various strain pairs. Legend: L – LOS locus; F – flagellar modification locus; C – capsular locus; R/M – restriction-modification locus.

As seen in Figure 3, the gene conservation profiles of some of these strain pairs showed a remarkable degree of congruence, with CGH profiles showing Pearson Correlation coefficients greater than 0.95 on the strength of similarities at multiple hypervariable loci. Each strain pair harboured subtle differences in gene content, ranging from 5 genes for the pair of GB25 and 98652 pair to 13 genes for the pair of EC023 and GC060, however, we did not identify any common gene content differences between the neuropathogenic and enteritis-control strains among the four strain pairs we examined [see Additional file 1].

## Conclusion

Current available evidence points to the direct involvement of genes that synthesize and transfer sialic acid to the LOS in the development of the cross-reactive anti-ganglioside antibodies thought to be the effectors in a large majority of GBS and MFS cases [25,26]. Although the association between neuropathogenic LOS genotypes and the GBS/MFS-associated phenotype is very strong, strains that bear the requisite genes for the synthesis of ganglioside mimics have been isolated from uncomplicated cases of enteritis. Similarly, a small number of GBS/MFS-associated strains do not appear to synthesize ganglioside mimics. Thus, the incomplete penetrance of the neuropathogenic LOS genotype has raised questions regarding the possible contribution of additional factors, whether bacterial or host-related, towards the development of these neuropathies. That the host contributes to such an auto-immune response should be considered likely.

A consistent finding among various genetic surveys of *C. jejuni *has been that GBS strains do not appear to represent a genetic lineage distinct from enteritis-control strains [12,14,38-40]. Studies attempting to correlate molecular typing results to the GBS/MFS phenotype did not reveal clusters of neuropathogenic strains distinct from enteritis-control strains, and results from our study suggest that many neuropathogenic strains are genomically related to enteritis-control strains. Some of the major clusters in the dataset include strains from more than one of the three major geographical regions represented in the study. For example, some HS:19 strains from The Netherlands and Curaçao cluster with, and are genetically similar to, strains from the Japanese HS:19 cluster. Similarly, HS:2 strains from The Netherlands cluster with strains from the two Japanese HS:2 lineages. It thus appears that the genomic stability previously suggested among clonal HS:19 strains of differing geographical source [38,41] is also valid among HS:2 strains, as has been suggested in other studies [42].

While cluster analysis is prominent in this study, the fact that neuropathogenic strains do not form a coherent cluster, and the fact that genes related to neuropathogenesis are expected to represent only a small fraction of the data, expose the shortcomings of the use of cluster analysis to define potential neuropathogenic markers. Because of this, in contrast to previous CGH-based studies [39,40], we opted to focus our search for neuropathogenic markers on the statistical testing of each individual gene present in the array for significant over-representation or under-representation among neuropathogenic strains. Using this novel approach, we were able to obtain unambiguous statistical signal for higher conservation rates in genes associated with LOS classes A and B among neuropathogenic strains [25,29,43]. These results not only agree with the current hypotheses regarding the mechanism of GBS/MFS but also concur with a recent study in which GBS/MFS strains and enteritis-control strains were screened for potential neuropathogenic markers using the high resolution comparative genomic method of high-throughput Amplified Fragment Length Polymorphism (htAFLP) [44]. This study revealed 3 markers highly-associated with GBS which mapped to the LOS locus.

The strong association observed for LOS classes A and B and neuropathogenic strains in our data was found despite the extensive genomic heterogeneity in these strains. A recent study has provided direct evidence for horizontal transfer of genes, including putative neuropathogenic factors in an experimental setting [45]. Our findings and the recent description of strain GB11, a GBS-associated HS:2 strain with high genetic similarity to the genome strain NCTC 11168 but found to have an A-class LOS locus instead of a C-class locus[36] would suggest that horizontal transfer represents an important mechanism for the dissemination of neuropathogenic factors between otherwise unrelated GBS/MFS strains.

Among the known neuropathogenic genes identified in our screen *cgtA *and *neuA1 *are known to be involved in LOS biosynthesis [24]. Another gene (*orf11*) has been found to be associated with class A/B LOS loci [25], and it was recently shown to encode a sialic acid acetyltransferase [46]. Genes in the LOS locus needed for the synthesis of ganglioside mimics are strong GBS/MFS determinants [25,26,29] but neuropathogenic strains with no known sialyltransferase genes are known to exist [25]. This raises the possibility, among others, that additional bacterial factors are required to elicit neurological complications. Even though we examined every gene in our microarray for similar associations with GBS/MFS strains, after filtering the data to avoid over- or under-representation due to clonality effects, only two additional genes (Cj1421c, Cj1428c or *fcl*) had statistically significantly higher conservation rates among neuropathogenic strains compared to enteritis-control strains; and a single gene (HS:3 Cj1135) had a higher conservation rate among enteritis-control strains. Cj1135 is a one-domain glucosyltransferase involved in LOS biosynthesis[47], while Cj1421c and Cj1428c are involved in capsule biosynthesis although their exact functions are still undefined [48]. The potential involvement of these three genes in neuropathogenesis, if any, needs to be assessed. At the same time, the potential involvement of additional bacterially-encoded factors should not be discounted.

The microarray used to generate the bulk of the data in this study includes known GBS-associated factors and comprises greater gene diversity than that used in a previously published study of GBS strains [39]. However additional genes important to neuropathogenicity could be missing from our array. This study represents the largest systematic screen for potential neuropathogenic factors in *C. jejuni *but anything less than a comparative approach involving full-genome sequences is only partially complete.

One of the key findings of this study is the close genetic relationship between some neuropathogenic strains and their enteritis-associated counterparts. This has been suggested by results from various molecular typing studies, and we have been able to observe these similarities with an extremely high level of resolution. Since several scenarios could help explain the high degree of genetic similarity observed between strains with different clinical outcomes, it is important to note that highly related strains can show major differences in virulence-associated phenotypes [49]. The LOS locus also presents a unique challenge in that diversity in LOS structures can be obtained through genetic variation affecting the relevant genes [24]. Enteritis-associated strains that carry neuropathogenic genes could have their neuropathogenic potential altered or silenced through mutation, and this mechanism is likely to play a significant role in the incomplete penetrance of the putative GBS/MFS genotype.

Our data suggest that in many cases GBS/MFS-related strains might not differ in their neuropathogenic potential with respect to highly genetically related enteritis-control strains. In some cases, differences in clinical outcome are likely to be attributable to differences in host-background. GB13 and GB14, two epidemiologically related strains isolated from a family outbreak in which only one of three individuals afflicted with enteritis went on to develop neurological complications [31], serve as a reminder of how host factors are likely to play a role in the development of neuropathic clinical outcomes. At the same time we have recently shown that GB11, a GBS strain with a close genetic relationship to the genome strain NCTC 11168, appears to have acquired potential GBS factors in the form of an A-class LOS locus [36]. That study clearly underscores the valuable insight that could be gained by comparing closely related strains with differing clinical outcomes in the discovery of potential neuropathogenic factors. Future efforts should be aimed at comparative-genomic sequencing of strain pairs such as the ones described in this study in order to address whether differences in pathogen or host are responsible for differences in the clinical outcome of Campylobacteriosis cases.

## Methods

### Bacterial strains and Genomic DNA Isolation

One-hundred and two strains were analyzed by microarray CGH (Table 2). The "Rotterdam Dataset" is comprised of 41 strains representing GBS, MFS, and enteritis-control isolates from The Netherlands and collected between 1990 and 1999 [12,14]. The "Curaçao Dataset" is comprised of 13 enteritis-control and GBS strains collected between 2000–2001 in the island of Curaçao (Netherlands Antilles) and is described in [50] and [51]. The "Dokkyo Dataset" is comprised of 48 strains collected from 1990–2003 in Japan and represents clinical isolates from enteritis-only, GBS, and MFS patients [52]. All bacterial cultures were initially grown on Karmali selective media (Oxoid) and subsequently on Mueller-Hinton agar plates (BACTO, Oakville, ON) for increased cell mass, (~24 hours at 37°C under microaerophilic conditions) prior to DNA isolation. Genomic DNA isolation was carried out as previously described [35].

**Table 2:** *Campylobacter jejuni *strains analyzed in this study

**Strains**	**Origin of isolate**	**Clinical outcome**	**HS serotype**	**No. of strains**
9xxx, 9xxxxx ^1^	The Netherlands	Enteritis	Various	19
GB ^2^	The Netherlands	GBS	Various	19
CURA ^3^	Curaçao*	Enteritis	Various	10
CURA/GB ^4^	Curaçao*	GBS	Various	3
MF ^5^	The Netherlands	MFS	Various	3
EC	Japan	Enteritis^6^	2	8
		Enteritis^7^	19	9
GC	Japan	GBS^8^	2	4
		GBS^9^	19	16
		MFS^10^	2	11

* Netherlands Antilles

^1 ^9072, 9123, 9126, 9138, 9140, 9141, 9144, 9146, 98623, 98652, 98706, 960094, 961089, 961090, 961095, 961163, 981087, 990520, 990521

^2 ^GB1, GB2, GB3, GB4, GB5, GB11, GB13, GB14, GB15, GB16, GB17, GB18, GB19, GB21, GB23, GB24, GB25, GB26, GB27

^3 ^CURA27, CURA29, CURA34, CURA40, CURA84, CURA112, CURA117, CURA170, CURA181, CURA235

^4 ^CURA276, GB22, GB28

^5 ^MF6, MF7, MF8

^6 ^EC23, EC26, EC43, EC55, EC56, EC68, EC73, EC97

^7 ^EC2, EC7, EC21, EC27, EC82, EC83, EC84, EC110, EC112

^8 ^GC114, GC147, GC150, GC177

^9 ^GC5, GC142, GC145, GC152, GC156, GC157 GC160, GC162, GC165, GC170, GC172, GC175, GC176, GC178, GC182, GC191

^10 ^GC20, GC21, GC39, GC51, GC57, GC60, GC68, GC124, GC125, GC155, GC164

### *C. jejuni *NCTC 11168 Open Reading Frame DNA Microarray

Details of the microarray, including primer selection, the parameters for primer synthesis, selection of amplicons, as well as the purification and printing of DNA onto slides were previously described elsewhere [35]. A new version of the microarray became available partway through this study. This new version incorporates additional genes not present in the genome strain NCTC 11168. Additional information is available at [53].

### Genomic DNA labelling

Genomic DNA was sheared into fragments ranging from 0.5 and 5 kilobases (mean size ~1.5 kilobases) using the method of Bodenteich *et al*. [54]. Briefly, genomic DNA was suspended in 35% glycerol and nebulized in an aerosol nebulizer (Medex, Carlsbad, CA, USA) for 45 seconds at 15 PSI. 5 μg of sheared DNA were fluorescently labelled using direct chemical coupling with the Label-IT (Mirus Corp., Madison, WI) cyanine dyes Cy3 and Cy5 as recommended by the manufacturer. Probes were purified from unincorporated dyes by sequentially passing samples through SigmaSpin (Sigma, Oakville, ON) and Qiaquick (Qiagen, Mississauga, ON) columns. Labelled DNA sample yields and dye incorporation efficiencies were calculated using the Nanodrop ND-1000 spectrophotometer (Nanodrop, Rockland, DE).

### Microarray hybridizations

The hybridization profile for each strain was obtained by co-hybridizing labelled DNA from the tester strain and from the NCTC 11168 (control) strain to our microarray. Equivalent amounts (1 to 2 μg) of labelled tester and control samples with similar dye incorporation efficiencies were pooled, lyophilized, and hybridized to microarrays as previously described [35].

### Microarray data acquisition and analysis

Microarrays were scanned using a Chipreader laser scanner (BioRad, Mississauga, ON) according to the manufacturer's recommendations. Spot quantification, visual inspection of potential outliers, and flagging of anomalous spots was performed using the program ArrayPro Analyzer (version 4.5; Media Cybernetics). The microarray data exported from ArrayPro was imported into the BioArray Software Environment (BASE version 1.2) [55] and is available at NCBI's Gene Expression Omnibus [56] under accession number GSE3579. Spots flagged due to poor spot morphology or low signal intensity (less than 5 × local background) were filtered out. After print-tip Loess normalization, data was used to calculate the average log ratio [log_2_(Signal Tester/Signal Control)] from the replicates for each gene represented on the microarray. The filtered data exported from BASE contains the average log ratio data for 1712 reporters. Log Ratio data was visualized and analyzed in TIGR's MultiExperiment Viewer (MEV version 3.0) [57]. Clustering of samples based on log ratio profile similarities was performed by the average linkage hierarchical clustering method of Eisen *et al*. [58], as implemented in TMEV, using Pearson correlation coefficient as a distance metric. The Support Tree method of bootstrapping implemented in TMEV was used to test the reliability of the clustering patterns (500 bootstrap re-samplings). The percentage of re-sampled trees supporting a given tree node are shown. To facilitate tree topology visualization, tree information was coded into Newick format and the trees were visualized using Treeview (version 1.6.6) [59].

### Statistical testing of gene conservation rates

For statistical analysis of differential Log Ratio averages between groups of isolates we used the T-test implemented in TMEV, using a modified Bonferroni-corrected significance threshold of P < 0.05. For statistical analysis of differential gene conservation rates between groups of strains, gene conservation profiles were obtained from Log Ratio data by categorizing genes into "present", "divergent", and "absent" according to thresholds that were empirically determined previously [35,37] To determine over- or under-representation of each gene among neuropathogenic isolates, the number of strains in which the gene was "present" and "absent"were calculated for representative groups of 32 neuropathogenic isolates and 32 enteritic isolates. P-values were then calculated for each gene on the microarray using the two-tailed Fisher's Exact test using an Microsoft Excel script developed in-house. Statistical significance of raw p-values was assessed using a threshold of P < 0.05; p-value adjustments were also performed to account for multiple testing using an in-house Microsoft Excel script that adapts the Westfall and Young permutation method to the gene conservation rate calculations described above [60]. Statistical results for the 169 genes displaying differences in conservation rates between neuropathogenic and enteritic groups are provided as Additional file 1.

## Abbreviations

GBS, Guillain-Barré syndrome;

MFS, Miller Fisher syndrome; 

LOS, Lipo-oligosaccharide; 

CGH, comparative-genomic hybridization.

## Competing interests

The author(s) declares that there are no competing interests.

## Authors' contributions

ENT designed M-CGH experiments, carried out downstream data analysis, and drafted the manuscript. RRA performed hybridizations, performed preliminary data analysis and assisted with downstream data analysis. MK characterized the Japanese strains. PCRG characterized the Dutch and Curaçao strains. JHEN, MG, and AvB conceived of the study, and participated in its design and coordination and helped to draft the manuscript. HPE, and NY participated in the conception and supervised the design of the study. All authors submitted comments on drafts and read and approved the final manuscript.

## Supplementary Material

Additional file 1 Statistical assessment of genes displaying differential conservation rates among enteritic and neuropathogenic isolates. The table represents the statistical assessment of the conservation rates among the genes that have been observed to be differentially conserved enteritic versus neuropathogenic isolates. Click here for file
